# Diagnostic and Prognostic Values of Cardiopulmonary Exercise Testing in Cardiac Amyloidosis

**DOI:** 10.3389/fcvm.2022.898033

**Published:** 2022-06-06

**Authors:** Rishika Banydeen, Astrid Monfort, Jocelyn Inamo, Remi Neviere

**Affiliations:** ^1^Clinical Research Department, CHU Martinique (University Hospital of Martinique), Fort de France, France; ^2^Cardiovascular Research Team EA7525, Université des Antilles (University of the French West Indies), Fort de France, France; ^3^Cardiology Department, CHU Martinique (University Hospital of Martinique), Fort de France, France; ^4^Cardiopulmonary Physiology Unit, CHU Martinique (University Hospital of Martinique), Fort de France, France

**Keywords:** cardiac amyloidosis, cardiopulmonary exercise testing, oxygen uptake, ventilatory efficiency, transthyretin (ATTR) amyloidosis, AL amyloidosis

## Abstract

Cardiac amyloidosis (CA) is a myocardial disease characterized by extracellular amyloid infiltration throughout the heart, resulting in increased myocardial stiffness, and restrictive heart wall chamber behavior. Its diagnosis among patients hospitalized for cardiovascular diseases is becoming increasingly frequent, suggesting improved disease awareness, and higher diagnostic capacities. One predominant functional manifestation of patients with CA is exercise intolerance, objectified by reduced peak oxygen uptake (VO_2_ peak), and assessed by metabolic cart during cardiopulmonary exercise testing (CPET). Hemodynamic adaptation to exercise in patients with CA is characterized by low myocardial contractile reserve and impaired myocardial efficiency. Rapid shallow breathing and hyperventilation, in the absence of ventilatory limitation, are also typically observed in response to exercise. Ventilatory inefficiency is further suggested by an increased VE-VCO2 slope, which has been attributed to excessive sympathoexcitation and a high physiological dead space (VD/VT) ratio during exercise. Growing evidence now suggests that, in addition to well-established biomarker risk models, a reduced VO_2_ peak is potentially a strong and independent predictive factor of adverse patient outcomes, both for monoclonal immunoglobulin light chain (AL) or transthyretin (ATTR) CA. Besides generating prognostic information, CPET can be used for the evaluation of the impact of therapeutic interventions in patients with CA.

## Introduction

Cardiac amyloidosis (CA) is a myocardial disease characterized by extracellular amyloid infiltration throughout the heart (1, 2). CA diagnosis among patients hospitalized for cardiovascular diseases is increasingly frequent, suggesting improved disease awareness and higher diagnostic capacities. Till now, only clinical and biomarker scoring systems have been accurately used to stratify disease severity in patients with CA (1, 2). In addition to these well-established risk models, it has been recently described that reduced peak aerobic capacity (VO_2_ peak) can help identify patients with poor prognoses (3–6).

The aim of the present review, addressed to clinicians and cardiologists, is to describe the utility of cardiopulmonary exercise testing (CPET) as an aid to patient orientation and clinical decision-making in CA management. Indeed, as patients with CA experience progressive exercise intolerance over their disease course, they will be increasingly referred for CPET evaluation. We outline here the role of CPET in assessing the functional status and prognosis in patients with CA, with a particular focus on ventilatory inefficiency.

## Clinical and Functional Evaluation of Patients With Cardiac Amyloidosis

Cardiac amyloidosis is a restrictive cardiomyopathy caused by the extracellular deposition of proteins in the myocardium (1, 2, 7, 8). The unstable structure of proteins causes them to misfold, aggregate, and deposit as amyloid fibrils. CA is caused mainly by misfolded monoclonal immunoglobulin light chains (AL) resulting from the abnormal clonal proliferation of plasma cells, or by misfolded transthyretin (TTR), a liver-synthesized protein (previously called pre-albumin) involved in hormone thyroxine and retinol protein binding and blood transportation. TTR can either be inherited as an autosomal dominant trait caused by pathogenic variants in the TTR gene (TTRv), or by the deposition of wild-type TTR protein (TTRwt) (7, 8). Cardiac involvement in systemic amyloidosis is suspected using electrocardiography, transthoracic echocardiography, as well as cardiac magnetic resonance and nuclear imaging. Confirmation of cardiac amyloid deposition is achieved through tissue biopsy, using thioflavin dyes (most commonly Congo red), displaying birefringence under cross-polarized light and conferring the characteristic apple-green color to amyloid deposits (1, 7, 8). Cardiac biomarkers are also helpful for risk stratification and staging of patients with AL and ATTR systemic amyloidosis (1, 2). Overall, CA is associated with a poor prognosis due to a high risk of sudden death, congestive heart failure, conductive disorders, and arrhythmias. One of the most frequent functional manifestations in patients with CA is exercise intolerance, which can be objectively evaluated by reduced exercise aerobic capacity.

## Central Hemodynamic Adaptation to Exercise in Cardiac Amyloidosis

The link between impaired exercise capacity and central hemodynamic alterations has been extensively studied in patients with CA (9–12). Typically, CA is predominantly considered a diastolic disease caused by increased myocardial stiffness and restrictive heart wall chamber behavior (9). Recent studies have also underlined a profound lack of myocardial contractile reserve and coronary flow velocity reserve (10–12). A blunt increase in stroke volume during exercise has been attributed to this decreased contractile reserve, rather than changes in preload and afterload (10). Impaired systolic myocardial performance during exercise has been further attributed to abnormal myocardial external efficiency, i.e., the ratio of left ventricle external stroke work to myocardial oxygen consumption (12). Impaired myocardial efficiency suggests that the coupling of myocardial oxidative metabolism to contractile work deteriorates, possibly due to structural amyloid damage to the heart, as well as cardiac cell toxicity in the case of AL amyloidosis (13, 14). Direct toxic effects of ATTR amyloid on cardiomyocytes have also been reported, such as oxidative stress, altered calcium cycling, and apoptotic pathways (15).

## Characteristics of Cardiopulmonary Exercise Testing in Cardiac Amyloidosis

Cardiopulmonary exercise testing with gas exchange determination not only enables peak oxygen uptake (VO_2_ peak) to be determined, but also sheds light upon the mechanisms of exercise curtailment, such as cardiovascular, ventilatory, and metabolic/muscular limitations (16). Peak exercise aerobic capacity (VO_2_ peak) is defined as the maximum ability of the cardiovascular system to deliver oxygen (O_2_) to exercising skeletal muscle, along with the ability of the skeletal muscle to maximally extract O_2_ from the blood (16, 17). As a result, exercise tolerance is determined by adequate cardiovascular performance, pulmonary gas exchange, and skeletal muscle metabolism. Key parameters estimated during CPET include oxygen uptake (VO_2_), pulmonary elimination of carbon dioxide (VCO_2_), minute ventilation (V_*E*_), as well as changes in cardiovascular parameters, such as heart rate and systemic arterial pressure rise (16, 17). Exercise intolerance in patients with CA was first demonstrated in case reports, describing symptoms of exertional syncope (18). Since then, severely reduced aerobic capacity (VO_2_ peak) in CA has been objectively demonstrated using the 6-minute walk test and CPET (3–6, 10, 12, 19–23). Table 1 summarizes the main CPET characteristics of patients with CA, which include reduced VO_2_ peak (expressed as low absolute peak values and anaerobic threshold VO_2_), increased V_E_-VCO_2_ slope, and episodes of oscillatory ventilation (EOV). The ventilatory response to exercise in these patients is further characterized by hyperventilation with rapid shallow breathing, in the absence of ventilatory limitation as evidenced by a normal ventilatory reserve (Table 1). A common additional feature of patients with CA is chronotropic incompetence, defined as insufficient cardio-acceleration (typically less than 80–85%) at peak exercise (4, 24). While the increase in normal stroke volume during exercise is lost in most patients with CA, cardiac output rises are primarily dependent on heart rate elevation. Thus, chronotropic incompetence may be considered a critical factor limiting exercise capacity in these patients. However, before concluding that a patient has chronotropic incompetence, it is important to consider the level of achieved effort and reasons for effort termination. Low exercise intensity will equal low metabolic stress and insufficient heart rate rise without true chronotropic incompetence. Chronotropic incompetence can be objectively demonstrated by the calculation of the slope between heart rate rise and VO_2_ increase during exercise. Using this metabolic-chronotropic relationship approach, no evidence of chronotropic incompetence was found in patients with ATTR CA (22).

**TABLE 1:** Main characteristics of cardiopulmonary exercise testing (CPET) in patients with cardiac amyloidosis (CA).

Characteristics	Trikas et al. (19)	Clemmensen et al. (10)	Hein et al. (3)	Clemmensen et al. (12) European heart	Yunis et al. (20)	Bartolini et al. (21)
CA type	AL	AL, ATTRwt, ATTRv	AL, ATTRwt, ATTRv	AL, ATTRwt, ATTRv	ATTRwt	ATTRwt, ATTRv
Number of patients	32	24	27	25	56	72
Age, years	50 ± 13	67 ± 15	58 (IQR 10)	69 [55–78]	75 ± 6	78 ± 8
LV wall thickness, mm	12 ± 3	18 (IQR 6)		16 ± 5	16 ± 3	18 ± 3
LVEF, %	32 ± 6	59 [45–63]	55 ± 28	54 ± 13	50 ± 11	54 ± 9
Watts peak, watts	–	75 [30–100]	–	–	–	–
VO_2_ peak, mL/kg/min *or* VO_2_ peak mL/kg (*)	21 ± 7	15 ± 6	15 (IQR 10)	1156 ± 496 *	14 ± 5	14 ± 4
Heart rate peak, bmp		125 [105–141]		123 ± 27		
ATVO_2_ mL/kg/min	13 ± 4				12 ± 5	
Peak RER	1 (IQR 0.1)				1 ± 0.1	
O_2_ pulse, percent						
V_E_VCO_2_ slope			30 (IQR 3)		41 ± 10	31 ± 7
EOV, % of cases						7
Ventilatory Reserve, %					43 ± 16	

**Characteristics**	**Monfort et al. (22)**	**Bhutani et al. (23)**	**Dalia et al. (5)**	**Nicol et al. (4)**	**Banydeen et al. (6)**

CA type	ATTRv	AL, ATTR	ATTRwt	AL, ATTRwt, ATTRv	AL, ATTRwt, ATTRv
Number of patients	18	17	33	150	46
Age, years	72 ± 8	64 [57–69]	82 [79–84]	70 [64–78]	72 ± 11
LV wall thickness, mm	20 [16–21]		16 [14–18]	15 [13–18]	16 ± 3
LVEF, %	49 [34–58]	63 [58–70]	50 [40–55]	55 [45–61]	54 ± 15
Watts peak, watts	65 ± 20			65 [50–90]	57 ± 20
VO_2_ peak, mL/kg/min	14 ± 4	13 [12–15]	11 [9–14]	13 [10–17]	16 ± 4
Heart rate peak, bmp	130 ± 19		110 [94–119]	117 [100–134]	
ATVO_2_ mL/kg/min	10 ± 3		8 [6–9]	8 [7–11]	
Peak RER	1 ± 0.1		1 [1.06–1.24]		1 ± 0.1
O_2_ pulse, %	62 ± 19			8 [7–10]	76 ± 17
V_E_VCO_2_ slope	39 ± 4		36 [31–41]	37 [33–45]	38 ± 6
EOV, % of cases	0				0
Ventilatory Reserve, %	27 ± 17				32 ± 19

AL, monoclonal immunoglobulin light chain amyloidosis; ATTRwt, wild type transthyretin amyloidosis; ATTRv, hereditary transthyretin amyloidosis; LV, left ventricle; LVEF, left ventricular ejection fraction; VO_2_, oxygen uptake; AT, anaerobic threshold; RER, respiratory exchange ratio; EOV, exercise oscillatory ventilation. Results are presented as mean ± SD, median and interquartile (IQR range, defined as the difference between the upper and lower quartiles) or median [Quartile 1–3]. * Specifies when VO2 peak is expressed in ml/kg.

## Prognostic Value of VO_2_ Peak in Patients With Cardiac Amyloidosis

**FIGURE 1:**
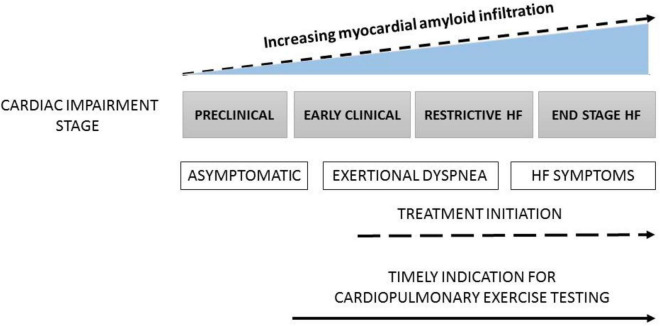
The progression of CA from asymptomatic stage to end stage heart failure (HF) and timing for the use of cardiopulmonary exercise testing (CPET).

In patients with heart failure, VO_2_ measured at peak exercise is the most important predictor of prognosis (25, 26). A VO_2_ peak of less than 12–14 ml/kg/min is associated with a poor outcome. Of note, the predictive value of the VO_2_ peak is accurate only when exercise capacity is limited by heart failure, which means that factors prematurely terminating the test must be excluded. Such factors include peripheral muscular deconditioning, peripheral artery disease, myocardial ischemia, or low patient motivation. Besides VO_2_ peak, parameters derived from the ventilatory response are also useful to determine risk in patients with heart failure. The ventilatory equivalent for carbon dioxide (V_E_/VCO_2_ ratio) and ventilatory efficiency (V_E_-VCO_2_ slope) are well-established indicators of prognosis (25–27). In patients with CA, growing evidence suggests that VO_2_ peak, along with usual risk scores, are strong and independent predictive factors, independently of CA etiology (AL or ATTR) (3–6, 10, 12, 19–23) (Figure 1). Overall, these findings are fairly consistent with the large body of evidence upholding the added value of VO_2_ peak in terms of efficacious risk stratification and outcome prediction in patients with heart failure. This constitutes a strong ground to believe that future multicenter studies will soon be confirming the prognostic capacity of CPET in patients with ATTR CA.

## Exertional Ventilatory Inefficiency in Patients With Chronic Heart Failure

In patients with known heart failure, ventilatory inefficiency, suggested by an increased V_E_-VCO_2_ slope, characterizes those with more severe heart disease and is considered an independent marker of event-free survival (28, 29). Interestingly, one of the most striking findings in patients with CA is an increased V_E_-VCO_2_ slope (Table 1). Of note, the latter is observed even in the absence of severe myocardial dysfunction, as suggested by the mild reduction in left ventricular ejection fraction (Table 1).

**FIGURE 2:**
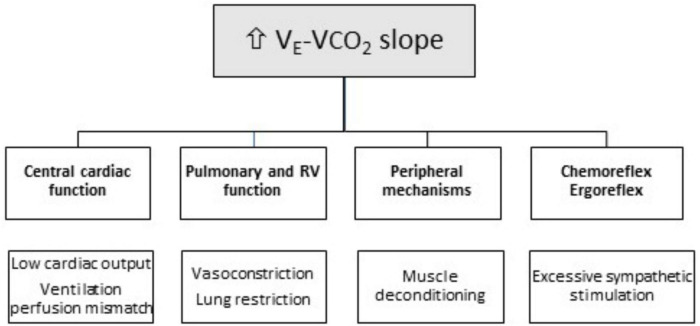
Mechanisms of ventilatory inefficiency (increased VE-VCO_2_ slope) in patients with cardiac amyloidosis (CA). RV, Right Ventricle.

The physiological determinants and underlying major mechanisms of ventilatory efficiency in patients with chronic heart failure and CA are presented next (Figure 2). The ramp with which ventilation rises with increased CO_2_ production, i.e., V_E_-VCO_2_ slope, reflects ventilatory efficiency (30, 31). Increased V_E_-VCO_2_ slope identifies an abnormal ventilatory response to exercise and has been consistently used as a prognostic marker in several chronic cardiopulmonary diseases, independently of other CPET-derived variables, such as VO_2_ peak. V_E_-VCO_2_ slope is calculated off-line after exercise using a linear regression function, and is established by the formula: $V\,E=\frac{863}{P\,a\,C\,O\,2\times \left(1-\frac{V\,D}{V\,T}\right)}\times V\,C\,O\,2$, where the factor 863 accounts for corrections related to body temperature, ambient pressure, water vapor saturated conditions (BTPS), VCO_2_ is the rate of CO_2_ pulmonary elimination, PaCO_2_ is arterial CO_2_ partial pressure, and V_D_/V_T_ is the physiological dead space ratio (30, 31). This formula predicts that V_E_-VCO_2_ slope is determined by two factors: (1) the direction and magnitude of PaCO_2_ change and (2) the fraction of the tidal volume that goes to dead space, i.e., the physiological dead space ratio (V_D_/V_T_). Therefore, V_E_-VCO_2_ slope increases if V_D_/V_T_ ratio remains elevated during exercise, or if PaCO_2_ is reduced.

Indeed, in patients with heart failure, V_D_/V_T_ ratio typically remains high during exercise due to increased heterogeneity of pulmonary ventilation-to-perfusion (V_*A*_/Q) ratios and/or blunted tidal volume increase V_T_ (30, 31) (Figure 2). In these patients, true V_*A*_/Q mismatch is uncommon, in the absence of coexisting lung disease. As such, while ventilation increases during exercise, cardiac output does not proportionally rise, which tends to augment the V_*A*_/Q ratio, resulting in a higher V_D_/V_T_ ratio and an increased V_E_-VCO_2_ slope. Similarly, patients with chronic heart failure often display a reduced V_T_ (volume of air moved into or out of the lungs during a normal breath) during exercise, which may also contribute to a higher V_D_/V_T_ ratio and increased V_E_-VCO_2_ slope (30, 31).

As for PaCO_2_, the other factor influencing V_E_-VCO_2_ slope, its reduction during exercise results in a rise in V_E_-VCO_2_ slope (30, 31). The point at which PaCO_2_ is set is determined by factors, such as metabolic acidosis, hypoxemia, baroreceptors in the pulmonary vasculature, and sympathetic nervous system hyperactivity. The PaCO_2_ set-point is driven down with excessive ventilatory stimulation from sensitized peripheral chemoreceptors and baroreceptors, or by ergoreceptors in the skeletal muscle. In patients with chronic heart failure, sympathetic activity is overstimulated and ventilatory responses to hypercapnia and hypoxia are exacerbated (32) (Figure 2). Furthermore, PaCO_2_ either remains stable or modestly declines from rest to peak exercise (31, 32). However, due to chemoreceptor hypersensitivity, even minor PaCO_2_ changes can elicit excessive hyperventilation and V_E_-VCO_2_ slope elevation (31, 32).

## Exertional Ventilatory Inefficiency in Patients With Cardiac Amyloidosis

Mechanisms leading to V_E_-VCO_2_ slope rise in patients with CA (Figure 2) have not been extensively studied. Autonomic dysfunction, along with sympathoexcitation and abnormal ventilatory patterns, may explain V_E_-VCO_2_ slope increase in patients with CA (33, 34). Amyloid disorders preferentially involve small unmyelinated nerve fibers, i.e., thinly myelinated Aδ and unmyelinated C fibers, and manifest with sensory loss mainly affecting pain and temperature sensation, as well as autonomic dysfunction (35, 36). The latter, a distinctive feature of patients with CA, is characterized by exaggerated sympathoexcitation and attenuated parasympathetic tone (37, 38). Cardiac dysautonomia manifests with altered heart rate variability and blood pressure regulation, arrhythmias or heart block, and autonomic (sympathetic) denervation (33, 34, 37–39). It is plausible to consider that sympathoexcitation may enhance the ventilatory response of both peripheral and central chemoreflexes, thus leading to enhanced ventilation and an increased V_E_-VCO_2_ slope (31, 32).

In patients with CA, right ventricular dysfunction is also becoming increasingly recognized. It is a well-known mechanism that results in an increased V_D_/V_T_ ratio and V_E_-VCO_2_ slope. Impaired pulmonary blood flow due to right ventricular dysfunction increases V_D_/V_T_ ratio, as regions of ventilated lung remain under perfused at rest and during exercise. A significant correlation between right ventricular dysfunction and V_E_-VCO_2_ slope has been reported in patients with CA, in accordance with similar findings in patients with chronic heart failure, in whom an increased V_E_-VCO_2_ slope also results from advanced right-sided heart dysfunction and impaired pulmonary hemodynamics.

Another mechanism that may contribute to increased V_D_/V_T_ ratio and V_E_-VCO_2_ slope in patients with CA is the absence of tidal volume V_T_ increase during exercise (32). While ventilatory reserve at peak exercise in patients with CA is typically unaffected, typical manifestations are rapid shallow breathing patterns characterized by low V_T_ and increased breathing frequency (6, 22). The inability to increase O_2_ delivery to respiratory muscles, translating into reduced respiratory muscle strength, and enhanced central and peripheral chemoreflexes, may explain the absence of tidal volume V_T_ rise during exercise (31, 32). Accordingly, we have shown that patients with CA commonly display a restrictive lung disease characterized by reduced lung volumes (6, 22). In this setting, reduced lung compliance, along with increased elastic loading on respiratory muscles, lung elastic recoil, and stimulated thoracic mechanoreceptors, limit the rise in V_T_ below 50–60% of maximal lung vital capacity, leading to a characteristic shallow breathing pattern with a high respiratory frequency (40).

## Changes in Exercise Tolerance After Treatment for Cardiac Amyloidosis

Treatments for CA vary depending on the type of amyloid. They include heart failure supportive therapy, therapies inhibiting the production of amyloid precursor proteins, and novel strategies inhibiting amyloid fibril formation, amyloid deposits, or stabilizing precursor proteins. In AL amyloidosis, the aim of treatment is to eradicate the underlying plasma cell clone that produces excess light chains (41). Chemotherapy regimens and autologous stem cell transplantation have been adapted from treatment of multiple myeloma, a related plasma cell dyscrasia. Well-established risk stratification dictates treatment strategy for patients with AL (42). Until recently, treatment of TTR CA was aimed at managing symptoms and disease-related complications. Novel therapies for the treatment of TTRwt and TTRv CA have since emerged. Over the past decade, different molecules targeting specific steps of the amyloidogenic cascade have been evaluated as potential drug candidates contributing to modified exercise tolerance in patients with TTR CA (43) (Table 2). Tafamidis, which binds to TTR and prevents tetramer dissociation and amyloidogenesis, has been shown to lower all-cause mortality at 30 months, as well as the deterioration rate during the 6-min walk test (44). Further investigations in patients with TTR CA have confirmed the beneficial effects of tafamidis on exercise capacity, associated with improved cardiac function and a reduction in myocardial amyloid deposition (45). Small interfering RNA or antisense oligonucleotide technologies are also highly effective for the blockade of TTR liver expression. While not a primary objective of randomized controlled studies (Table 2), the analysis of cardiac parameters in hereditary CA cohorts has suggested a beneficial effect on the progression of cardiomyopathy. Indeed, preliminary data suggest that inotersen, a second-generation antisense oligonucleotide targeting TTR messenger RNA, can improve exercise tolerance and reduce left ventricular mass over a 3-year treatment period (46). Large clinical trials involving RNA-targeting and gene editing therapies are currently underway and will probably confirm the beneficial effects of these novel treatments on exercise tolerance in patients with TTR CA (43).

**TABLE 2:** Main studies evaluating treatment for transthyretin (TTR) CA with functional end-points.

	ATTR silencers		ATTR stabilizers
Molecule	Patisiran siRNA LNP	Inotersen 2′- MOE modified ASO	Tafamadis
Study	APOLLO 2018 randomized controlled trial (47) 225 patients ATTRv polyneuropathy	NEURO-TTR 2018 randomized controlled trial (48) 172 ATTRv polyneuropathy patients, 105 (61%) with cardiac involvement	ATTR-ACT study (44) 441 ATTR-CA patients (106 ATTRv-CA and 335 ATTRwt-CA)
Main results	Patisiran significantly improved neuropathy scores, QOL, walking parameters, nutritional status, and activities of daily living	Inotersen modified the course of neuropathy and improved QOL	Tafamidis was associated with a reduction in all-cause mortality and cardiovascular hospitalizations
Functional evaluation	In a pre-specified ATTRv-CA subgroup, Patisiran improved functional capacity (10-m walk test)	In an interim analysis, 33 patients with ATTR-CA treated with inotersen had decreased LV mass and improved exercise tolerance in 6MWT (46)	Secondary end points were notable for a lower rate of functional capacity decline (6MWT distance) and of quality-of-life decline
Future studies with functional end-points	APOLLO B https://ClinicalTrials.gov/show/NCT03997383 Evaluate Patisiran for ATTRv-CA and ATTRwt-CA with the primary end point of 6-minute walk test (6MWT) performance and secondary end points of death and hospitalization at 12 months.		

*2′- MOE, 2′- O- methoxyethyl; ASO, antisense oligonucleotide; ATTR, amyloid transthyretin; ATTRwt, wild type transthyretin amyloidosis; ATTRv, hereditary transthyretin amyloidosis; LV mass, Left Ventricular mass; ATTR-ACT, Safety and efficacy of tafamidis in patients with transthyretin cardiomyopathy; ATTR-CA, transthyretin amyloidosis with cardiomyopathy; QOL, quality of life; 6MWT, 6-min walk test.*

## Future Prospects

Cardiac involvement is the major negative prognostic factor, as deaths are mainly due to heart failure or arrhythmias associated with amyloidosis. Diagnostic strategies for symptomatic CA, presenting with typical morphological features, such as increased ventricular wall thickness, are well established. On the other hand, detection of subclinical CA is much more limited. At a time when effective therapies suppressing or delaying CA progression are available, the early detection of non-clinically overt forms of the disease is more than ever warranted. A large number of extra-cardiac symptoms, called “red flags,” can direct toward CA investigation and detection in its sub-clinical phase. Indeed, from a patient’s perspective, many other extra-cardiac symptoms are typically reported, such as breathlessness, tingling sensation, pain, difficulty in walking, and weight loss.

In the same line of thought, CPET, while not a diagnostic tool, can be extremely useful for the detection of early stages of cardiovascular and lung diseases, as well as for the evaluation of response to therapies (Figure 1). Indeed, it is today quite established that CPET can non-invasively identify early hemodynamic and metabolic alterations in patients with various cardiomyopathies, long before the latter become clinically overt and cardiac structural changes are already in place. In CA, CPET should be used to detect early functional involvement of the disease.

## Conclusion

Patients with CA display exertional dyspnea and exercise intolerance, evidenced mainly by poor aerobic capacity (reduced VO_2_ peak) and ventilatory inefficiency (increased V_E_-VCO_2_ slope). Both impaired VO_2_ peak and increased V_E_-VCO_2_ slope, two key parameters estimated during CPET, have been associated with poor outcome, both in monoclonal immunoglobulin light chain (AL) and transthyretin (ATTR) CA. These findings quite strongly advocate for the consideration of CPET, alongside traditional clinical and biological risk models at CA diagnosis, in order to optimize patient risk stratification. Ongoing large multicenter studies are currently evaluating the value of CPET in the prognostic assessment of patients with systemic amyloidosis. In time, CPET should become an integral part of CA management, contributing toward the assessment of the impact of novel therapies on the functional capacity and quality of life of patients with CA.

## Author Contributions

AM and RB collected the literature data. JI and RN analyzed and interpreted the literature data. RN, RB, and AM wrote the manuscript. All authors contributed, read, and approved the final manuscript.

## Conflict of Interest

The authors declare that the research was conducted in the absence of any commercial or financial relationships that could be construed as a potential conflict of interest.

## Publisher’s Note

All claims expressed in this article are solely those of the authors and do not necessarily represent those of their affiliated organizations, or those of the publisher, the editors and the reviewers. Any product that may be evaluated in this article, or claim that may be made by its manufacturer, is not guaranteed or endorsed by the publisher.
